# Tissue Doppler derived biphasic velocities during the pre and post-ejection phases: patterns, concordance and hemodynamic significance in health and disease

**DOI:** 10.1186/s12947-022-00287-0

**Published:** 2022-07-14

**Authors:** Alaa Mabrouk Salem Omar, Diana Maria Ronderos Botero, Javier Arreaza Caraballo, Ga Hee Kim, Yeraz Khachatoorian, Jaclyn Kliewer, Mohamed Ahmed Abdel Rahman, Osama Rifaie, Jonathan N. Bella, Edgar Argulian, Johanna Contreras

**Affiliations:** 1 Department of Cardiology, Mount Sinai Morningside, 1111 Amsterdam avenue, NY 10025 New York, USA; 2 Department of Cardiology, Icahn School of Medicine at Mount Sinai, New York, NY USA; 3 Department of Internal Medicine, BronxCare Hospital Center, Bronx, NY USA; 4 Department of General Surgery, HCA Florida Kendall Hospital, Miami, Florida USA; 5 Depratment of Cardiology, Ain Shams University Hospital, Cairo, Egypt

**Keywords:** Heart failure, Tissue doppler imaging, Pre-ejection, Post-ejection

## Abstract

**Background:**

Pre-(PRE) and post-ejection (POE) velocities by mitral annular tissue Doppler (TD) are biphasic and may be related to myocardial deformations. We investigated the predominance and concordance of TD-PRE and POE velocities and their effect on myocardial functions in controls and in heart failure (HF) patients.

**Methods:**

Retrospectively, 84 HF patients [57.6 years, 28(33%) females, NYHA: 2.3 ± 0.6, EF: 55 ± 15%, 52(62%) preserved EF, and 32(38%) reduced EF], 42 normal young controls, and 26 asymptomatic age matched controls were included. Echocardiography was done and from mitral annular tissue Doppler recordings, the biphasic PRE and POE velocity signals were identified and compared between groups.

**Results:**

While controls had almost always predominantly positive PRE and negative POE, HF had more negative PRE and positive POE. Moreover, almost all controls exhibited normal concordance (positive PRE and negative POE). HF exhibited more abnormal concordance which was significantly associated with worse NYHA, and parameters of diastolic and systolic functions. Opposite PRE and POE velocities correlated significantly in all groups (PREp vs POEn: young:r = 0.52, *p* < 0.001, age controls:r = 0.79, *p* < 0.001, HFpEF: r = 0.56, *p* < 0.001, HFrEF: r = 0.42, *p* = 0.018; PREn vs POEp: young: r = 0.25,*p* = 0.1, age controls: r = 0.42, *p* = 0.04, HFpEF: r = 0.43, *p* = 0.004, HFrEF: r = 0.61, *p* < 0.001) and the ratios PRE-P/N and POE-N/P correlated significantly with E/e’ in HF only.

**Conclusions:**

In physiological state, TD signals are predominantly positive during PRE and negative during POE. Opposite PRE and POE velocities corelate, representing the PRE-generation and POE-reversal of shortening–stretch relationships, the attenuation of which in HF may be related to elevated LV filling pressures. In HF, partially or completely reversed concordance of PRE and POE is associated with progressive worsening of clinical and hemodynamic profiles.

## Background

Heart failure (HF) is a leading cause of death worldwide, the incidence and the prevalence of which is only expected to increase especially in the face of an aging population [1]. Several classifications for heart failure exist, the most acceptable of which is based on left ventricular (LV) ejection fraction (EF) into HF with preserved EF (HFpEF) and reduced EF (HFrEF), and based on functions into systolic HF and diastolic HF [2]. Regardless of the type of classification, current models fail to encompass the mechanistic behaviors of myocardial structure and function in health and disease. For instance, despite the intuitive separation between systole and diastole as two consecutive phases of the cardiac cycle, it is impossible to separate them functionally. Surprisingly, large lack of understanding exists for the pre-ejection and post-ejection phases despite that these brief periods of the cardiac cycle are crucial for pressure buildup and loss that are essential for systolic ejection and diastolic filling, respectively.

Velocities during pre-ejection (PRE) and post-ejection (POE) recorded by tissue Doppler imaging (TDI) of the mitral annulus are biphasic. Several studies have shown that the positive velocity during PRE (PREp) represents a less load independent state of myocardial contractility and is associated with clinical and hemodynamic variables in patients with systolic dysfunction [3, 4].

Moreover, we have recently reported that PREp is associated with LV filling pressure in systolic dysfunction [5] and in patients with mitral regurgitation [6]. The biphasic nature of these velocities is, reportedly, related to myocardial deformation in the form of endocardial shortening and epicardial stretch in the PRE and their reversal during the POE [7]. These velocities and their expression in health and disease and their relationship to clinical and hemodynamic profiles in HF patients have not been well studied. In the current study, we have aimed to investigate the normal predominance of the biphasic velocities during PRE and POE phases and the PRE-POE biphasic velocity concordance in normal controls and in patients with heart failure and the effects of different concordance variations on myocardial functions in patients with HF.

## Methods

In a retrospective study protocol, patients referred to the echocardiography laboratory on outpatient basis with symptoms suggestive of heart failure were included. In addition, normal young controls (age < 40 years) and patients referred for routine echocardiographic examination who had no symptoms or structural myocardial abnormalities and who were age matched to heart failure patients were also included in the study (young controls and age matched controls, respectively). Patients were excluded if they had terminal illness, were in atrial fibrillation, had more than mild valvular disease, or had less than acceptable echocardiographic image quality. Demographic, clinical and laboratory variables were compared between groups and, in addition, conventional echocardiographic examination focused on tissue Doppler recording and measurements were also compared between subgroups.

### Echocardiographic examinations

All echocardiographic studies were performed with a commercially available echocardiography systems equipped with a 2.5-MHz phased array transducer. Digital routine grayscale 2-dimensional and tissue Doppler cine loops from 3 consecutive beats were obtained at end-expiratory apnea from standard apical views at depths of 12–20 cm. Sector width was optimized to allow for complete myocardial visualization while maximizing the frame rate. Gain settings were adjusted for routine clinical grayscale 2D imaging to optimize endocardial definitions. LV-EF was calculated from the apical 2- and 4-chamber images using the biplane Simpson’s technique. EF was defined as preserved if EF was > 50% and reduced if EF was ≤ 50%. All measurements were made in ≥ 3 consecutive cardiac cycles and average values were used for the final analyses. The pulsed-wave Doppler-derived transmitral velocity and spectral tissue Doppler-derived mitral annular velocity were obtained from the apical 4-chamber view. The early diastolic wave velocity (E) and the late diastolic atrial contraction wave velocity (A) were measured using pulsed-wave Doppler recording, and the early diastolic mitral annular velocity (e’) and systolic velocity (s’) were measured and averaged from the septal and lateral mitral annular positions. The E/e´ ratio was calculated to assess LV filling pressure (LVFP) for all patients.

### Pre and post-ejection tissue velocities: patterns, predominance and concordance

**Fig. 1:**
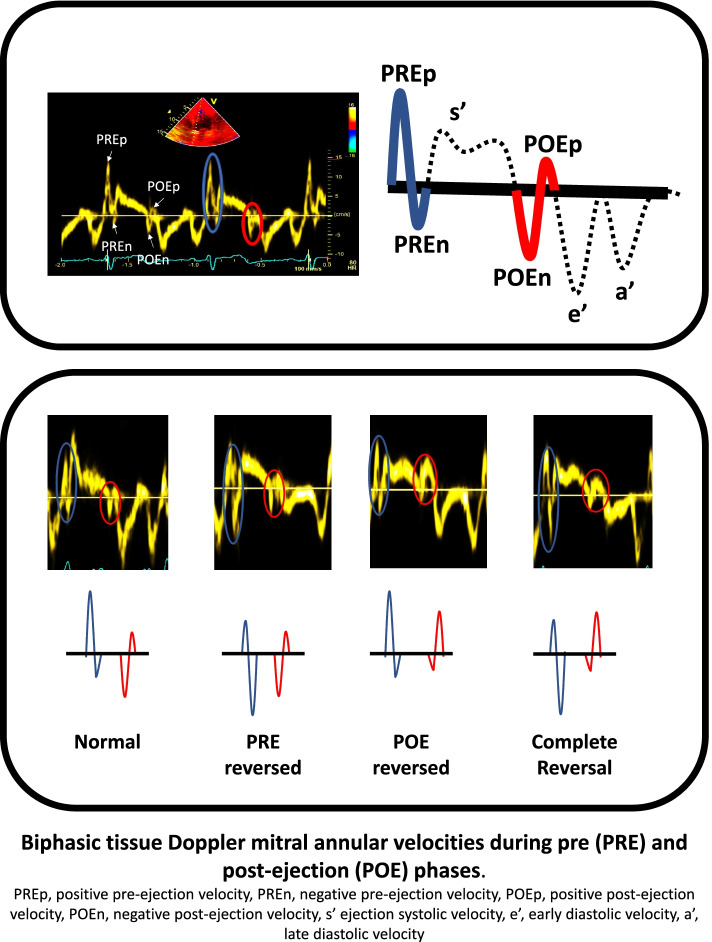
Biphasic myocardial velocities during pre and post-ejection phases in controls and heart failure patients. Upper panel: Tissue Doppler-derived as well as schematic representation of tissue Doppler (TDI)-derived mitral annular velocities in normal controls. In blue, the predominantly positive biphasic pre-ejection (PRE), the positive component (PREp) predominates over the following negative component (PREn). In red, the predominantly negative biphasic post-ejection (POE), the negative component (POEn) predominates over the following positive component (POEp). Lower panel, representation of different concordance of isovolumic phases in heart failure patients, from left to right, normal concordance, predominantly positive PRE and predominantly negative POE, reversed PRE concordance, predominantly negative PRE and POE, reversed POE concordance, predominantly positive PRE and POE, totally reversed concordance, predominantly negative PRE and predominantly positive POE

From the mitral annular tissue Doppler recordings, the biphasic pre-ejection (PRE) and post-ejection (POE) velocity signals were identified (Fig. 1). PRE was identified as a positive signal (PREp) followed by a negative signal (PREn) that preceded the ejection systolic velocity (s’). POE was identified as a negative signal (POEn) followed by a positive signal (POEp) that was positioned between the ejection systolic velocity (s’) and early diastolic velocity (e’). All positive and negative signals of both phases were measured and averaged from the septal and lateral mitral annular positions. The ratio of both PRE and POE velocities were obtained as chronologically occurring during the cardiac cycle as the former divided by the later in the form of the positive to negative PRE (PRE-P/N), and negative to positive POE (POE-N/P). The predominance of the biphasic PRE was defined as positive if PRE-P/N was > 1, and negative if PRE-P/N was ≤ 1. The predominance of the biphasic POE was defined as negative if POE-N/P was > 1, and positive if IR-N/P was ≤ 1. Next, the concordance of both biphasic velocities was defined as Normal if PRE was predominantly positive and POE was predominantly negative, reversed PRE, if both velocities were predominantly negative, reversed POE if both velocities were predominantly positive, and complete reversal if PRE was predominantly negative and POE was predominantly positive.

### Statistical methods

Categorical data are presented as number (%) and were compared using chi square test. Continuous data are presented as mean ± SD. Data were tested for normality using Kolmogorov–Smirnov and Shapiro–Wilk tests and, accordingly, continuous data were compared using t-test or analysis of variance (ANOVA) if they are normally distributed or the Mann–Whitney U test if they are not normally distributed. The correlations between different variables were done using the Pearson correlation coefficient. Cut-off values for prediction of E/e’ > 13 were tested using receiver-operator-characteristic (ROC) curves. Reproducibly analyses were done using interclass correlation coefficient and absolute differences for inter-observer and intra-observer differences of repeated measures in 15 randomly selected patients. Differences were considered statistically significant at *p* < 0.05. All analyses will be performed with a commercially available software (SPSS, version 23.0; SPSS, Inc).

## Results

The initial study group included 102 patients. 18(18%) patients were excluded due to atrial fibrillation in 5(5%) patients, and significant mitral valve disease in 13(13%) patients [6 patients with significant rheumatic mitral stenosis, and 7 patients with moderate to severe mitral regurgitation due to leaflet tethering], Accordingly, the final study cohort consisted of 84 heart failure patients [age: 57.6 ± 8 years, 28(33%) females, NYHA: 2.3 ± 0.6, EF: 55 ± 15%]. Of these, 52(62%) patients had HFpEF (EF: 65 ± 5%), and 32(38%) patients had HFrEF (EF: 39 ± 9%). The cause of heart failure in patients with HFpEF was related to hypertension in 18 patients, diabetes in 11, both hypeertension and diabetes in 14 patients, and coronary artery disease in 9 patients, and the cause of heart failure in patients with HFrEF was ischemic cardiomyopathy in 9 patients, and non-ischemic cardiomyopathy in 23 patients. In addition, 42 young normal subjects [age: 27 ± 4 years, 5(12%) females, EF: 66 ± 5%], and 26 age matched controls [age: 53 ± 12 years, 28(65%) females EF: 61 ± 4.7%] were included. Table 1 shows comparisons between all groups regarding baseline demographic clinical and echocardiographic variables. Briefly, patients with HFpEF were the oldest and had more risk factors, while patients with HFrEF had worse systolic and diastolic echocardiographic parameters.

**Table 1:** Baseline demographic, clinical and echocardiographic characteristics in heart failure patients as well as normal and age matched controls

	**HFpEF (*n* = 52)**	**HFrEF (*n* = 32)**	**Normal (*n* = 42)**	**Age matched Control (*n* = 26)**	***p*-value**
Age, years	58.1 ± 8.2	56.7 ± 8	27.3 ± 3.95	53 ± 11.474	< 0.001 #‡€
Sex, (female), n(%)	19(37)	9(28)	5(12)	17(65)	< 0.001
Diabetic, n(%)	21(40)	11(34)	0(0)	0(0)	0.009
Hypertensive, n(%)	23(44)	12(38)	0(0)	8(31)	< 0.001
Hyperlipidemia, n(%)	8(15)	3(9)	0(0)	1(4)	0.292
Smoker, n(%)	14(27)	8(25)	0(0)	4(15)	0.037
Coronary artery disease, n(%)	17(33)	16(50)	0(0)	2(8)	< 0.001
PCI, n(%)	8(15)	9(19)	0(0)	1(4)	0.210
CABG, n(%)	0(0)	0(0)	0(0)	0(0)	1.000
NYHA (1/2/3/4), n(%)	4(8)/31(60)/15(29)/0(0)	1(3)/18(56)/11(34)/2(6)	-	-	
E (cm/s)	75 ± 27	88.7 ± 21.5	82.7 ± 16.7	71 ± 18.5	0.011^*^$
A (cm/s)	81.8 ± 20.7	71.2 ± 33.8	56 ± 8.1	76.2 ± 21.6	< 0.001^¶€^
EDV (ml)	99.2 ± 21.7	152.5 ± 54.3	95.4 ± 17.8	87.8 ± 19.3	< 0.001^*‡$^
ESV (ml)	35.4 ± 10.3	96.2 ± 49.8	32.2 ± 7.7	30.7 ± 10	< 0.001^*‡$^
EF (%)	65 ± 5.1	38.9 ± 9.2	66.3 ± 5.1	61.5 ± 4.7	< 0.001^*^‡$€
e'-septal (cm/s)	6.5 ± 1.9	5.6 ± 1.7	11 ± 2.4	8.6 ± 2.3	< 0.001^#¶‡$€^
S'-Septal (cm/s)	7.5 ± 1.5	5.6 ± 1.8	8.2 ± 1.6	8.27 ± 1.8	< 0.001^*‡$^
e'-lateral (cm/s)	8.4 ± 2.8	7.1 ± 2.5	14 ± 2.8	10.9 ± 2.8	< 0.001^*‡$€^
S'-lateral (cm/s)	7.9 ± 2	6.2 ± 1.8	10.1 ± 2	8.9 ± 1.9	< 0.001^*#‡$^
e'-mean (cm/s)	7.5 ± 2.2	6.3 ± 1.9	12.5 ± 2.5	9.8 ± 2.4	< 0.001^#¶‡$€^
S’-mean (cm/s)	7.7 ± 1.5	5.9 ± 1.5	9.2 ± 1.6	8.5 ± 1.6	< 0.001^*#‡$^
E/A	0.96 ± 0.45	1.41 ± 0.77	1.52 ± 0.41	0.96 ± 0.25	< 0.001^*#$€^
E/e'	10.7 ± 4.4	14.6 ± 4.55	6.63 ± 0.83	7.6 ± 1.73	< 0.001^*#¶‡$^
PREp (septal), (cm/s)	6.8 ± 2.2	4.5 ± 1.9	7.9 ± 2.3	7.8 ± 2.1	< 0.001^*‡$^
PREn (septal), (cm/s)	3.3 ± 1.6	3.1 ± 1.5	3 ± 1.3	3.1 ± 1.3	0.793
POEn (septal), (cm/s)	3.3 ± 1.1	2.6 ± 0.98	4.5 ± 1	4.2 ± 1.2	< 0.001^*#¶‡^$
POEp (septal), (cm/s)	2.6 ± 0.8	2.7 ± 1.5	1.9 ± 0.7	2.1 ± 0.6	0.002^#‡^
PREp (lateral), (cm/s)	5.7 ± 2.5	4.1 ± 1.4	6.8 ± 1.8	7.5 ± 2.4	< 0.001^*¶^‡$
PREn (lateral), (cm/s)	4 ± 1.2	3.5 ± 1.8	3.2 ± 1.4	3 ± 1.08	0.014^¶^
POEn (lateral), (cm/s)	3.3 ± 1.1	2.3 ± 0.8	4.8 ± 1.6	3.5 ± 1.1	< 0.001*^#‡$€^
POEp (lateral), (cm/s)	2.7 ± 0.8	2.9 ± 0.9	2.1 ± 0.7	2.1 ± 0.6	< 0.001^#¶‡^$
PREp (mean), (cm/s)	6.3 ± 2.2	4.3 ± 1.6	7.1 ± 1.7	7.2 ± 1.9	< 0.001^*‡$^
PREn (mean), (cm/s)	3.7 ± 1.1	3.3 ± 1.4	3.1 ± 0.96	2.9 ± 0.8	0.016^¶^
POEn (mean), (cm/s)	3.3 ± 1	2.4 ± 0.7	3.9 ± 1.4	3.7 ± 0.9	< 0.001^*#‡$^
POEp (mean), (cm/s)	2.6 ± 0.7	2.8 ± 0.99	2.6 ± 1.2	1.98 ± 0.63	0.005^¶^€
PREp/PREn (PRE-P/N)	1.8 ± 0.7	1.5 ± 1.03	2.5 ± 1.05	2.6 ± 0.7	< 0.001^#¶‡$^
POEn/POEp (POE-N/P)	1.3 ± 0.5	1.1 ± 1.04	2.2 ± 1.07	2.1 ± 1.3	< 0.001^#¶‡$^
PREp-mean/S’-mean	0.82 ± 0.3	0.7 ± 0.2	0.8 ± 0.2	0.9 ± 0.22	0.182
LBBB, n(%)(**)	1(2%)	7(22%)	0(0%)	0(0%)	0.519
RBBB, n(%)(**)	3(6%)	0(0%)	0(0%)	1(4%)	0.626
IVCD, n(%)(**)	0(0%)	4(13%)	0(0%)	0(0%)	0.041
QRS duration, ms	84.4 ± 12.7	96.2 ± 24	72.6 ± 11	79.2 ± 18	< 0.001^*‡$^

^*^ *p* < 0.05 between HFpEF and HFrE

^#^ *P* < 0.05 between HFpEF and normal

^¶^ *p* < 0.05 between HFpEF and Controls

^‡^ *p* < 0.05 between HFrEF and norma

^$^ *p* < 0.05 between HFrEF and Controls

^€^ *p* < 0.05 between normal and controls. PREp, positive pre-ejection, PREn, negative pre-ejection, POEn, negative post-ejection, POEp, positive post-ejection. PREp, positive pre-ejection, PREn, negative pre-ejection, POEn, negative post-ejection, POEp, positive post-ejection

### Pre and post-ejection phases in patients and controls

**Fig. 2:**
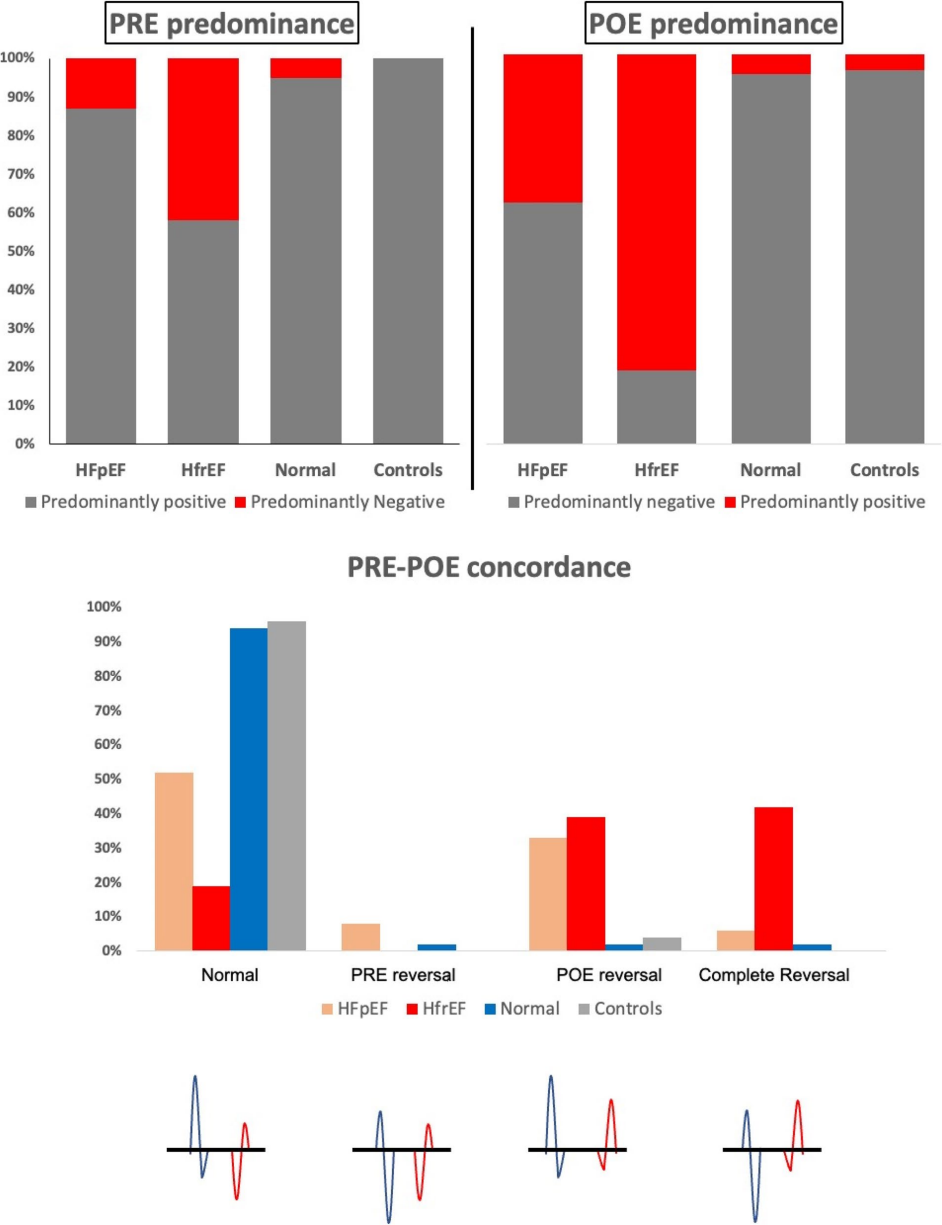
Frequency of predominance of biphasic tissue Doppler mitral annular velocities during pre (PRE) and post-ejection (POE) phases, and their concordance. Upper panel, in normal as well as age matched controls, PRE is almost always predominantly positive (PREp > PREn), while more patients with negative predominance (PREn > PREp) occurs in HFpEF and the largest number occurs in patients with HFrEF. Similarly, in normal as well as age matched controls, POE is almost always predominantly negative (POEn > POEp), while more patients with positive predominance (POEp > POEn) occurs in HFpEF and the largest number occurs in patients with HFrEF. Lower panel, Normal PRE-POE concordance (predominantly positive PRE and predominantly negative POE) occurs almost always in normal as well as the aged matched controls. In patients with HFpEF, lower number of normal concordance and increasing number of PRE reversed and POE reversed as well as totally reversed concordance was noted. In patients with HFrEF, the lowest number of normal concordance, and the largest number of totally reversed concordance was observed

Table 1 summarizes comparisons between patients and controls for septal, lateral, and averaged mitral annular velocities including pre-ejection (PRE) and post-ejection (POE) variables. It was found that, patients with heart failure had lower PREp, and POEn, higher PREn and POEp, and lower ratios PRE-P/N and POE-N/P compared to controls. Among heart failure patients, HFrEF tended to have lower PREp, and POEn compared to HFpEF (PREp: 6.3 ± 2.2 vs. 4.3 ± 1.6, *p* < 0.001; POEn: 3.3 ± 1 vs. 2.4 ± 0.7, *p* < 0.001) while there was no significant difference between PREn and POEp compared to HFpEF (PREn: 3.7 ± 1.1 vs. 3.3 ± 1.4, *p* = 0.225; POEp: 3.3 ± 1 vs. 2.4 ± 0.7, *p* = 0.248) and, similarly, there was no significant difference regarding both ratios (POE-P/N: 1.8 ± 0.7 vs. 1.5 ± 1.03, *p* = 0.282; POE-N/P: 1.3 ± 0.5 vs. 1.1 ± 1.04, *p* = 0.203). On the other hand, with the exception of a higher POEp in the normal subjects, none of the PRE or POE velocities were different between normal subjects and matched controls. Moreover, it was noted that normal and age matched controls had almost always a predominantly positive PRE and predominantly negative POE, while predominantly negative PRE and predominantly positive POE occurred almost always in HF patients (Fig. 2).

### Concordance between biphasic velocities of pre and post-ejection phases in patients and controls

Next, all patients and controls were compared based on our definition of concordance between both biphasic velocities (Normal: PRE predominantly positive and POE predominantly negative, reversed PRE: both PRE and POE predominantly negative, reversed POE: both PRE and POE predominantly positive, and complete reversal: PRE predominantly negative and POE predominantly positive, Fig. 2). It was found that almost all normal subjects and matched controls exhibited normal concordance [normal subjects: 39 (93%) subjects, and matched controls: 23(96%) patients], while in normal subjects, 3(7%) subjects exhibited abnormal concordance (reversed PRE in 1 reversed POE in 1, and total reversion in 1), and in matched controls one (4%) patient exhibited reversed POE.

Importantly, abnormal concordance was found in significantly more HF patients compared to controls [49(58%) patients vs. 4(6%) controls, *p* < 0.001]. Abnormal concordance occurred in 24(46%) HFpEF patients and 25(67%) HFrEF patients, (*p* = 0.003). All types of concordance were observed in patients with HFpEF, however, in patients with HFrEF, almost all patients had abnormal concordance [12(44%) reversed POE, and 13(48%) totally reversed concordance]. Importantly, concordance seemed to be significantly associated with NYHA functional class such that the best NYHA was in normal concordance (1.6 ± 1) and the worst was in reversed concordance (2.6 ± 0.5), while reversed PRE and POE was in between normal and reversed (2 ± 1.2 and 2.3 ± 0.6, respectively). Similar patterns were observed in parameters of diastolic and systolic functions where the highest E/A and E/e’, the lowest average e’, the largest ESV and EDV and the lowest EF were noted in reversed concordance, while all these parameters were noted to be best in normal concordance and were intermediate between both groups in reversed PRE and POE. Importantly, in heart failure patients, bundle branch block was not significantly different between groups, while intraventricular conduction delay was most frequent in patients with totally reversed concordance (Table 2). Moreover, electrocardiographic QRS duration was lowest in patients with normal concordance, larger in patients with reversed concordance among which it was largest in patients with totally reversed concordance (Table 2).

**Table 2:** Comparison of study subjects based on PRE-POE concordance

	**PREp and POEn (Normal, *n* = 96)**	**PREn and POEn (PRE reversed, *n* = 5)**	**PREp and POEp (POE reversed, *n* = 31)**	**PREn and POEp (Totally reversed, *n* = 18)**	***p*-value**
Age, years	46.7 ± 15.4	53 ± 17.9	57.4 ± 6.92	58.9 ± 9.6	< 0.001^#¶^
Sex, (female), n(%)	29	2	12	6	0.671
HFpEF/HFrEF/ Normal/Control, n(%)	28/6/39/23	4/0/1/0	17/12/1/1	3/13/1/0	< 0.001
NYHA (I/II/III/IV), n(%)	3/24/6/1	0/2/2/0	3/16/9/0	0/7/9/0	0.015
NYHA (mean ± SD)	1.6 ± 1	2 ± 1.2	2.2 ± 0.6	2.6 ± 0.5	0.001^#¶^
E (cm/s)	74.3 ± 19.1	95.4 ± 25.9	80.6 ± 23.2	97.5 ± 30.7	0.001^¶^
A (cm/s)	71.9 ± 22.7	68.3 ± 17.5	85.8 ± 25.1	62.1 ± 27.3	0.01^#€^
EDV (ml)	98.1 ± 24.6	113.7 ± 29.7	115.7 ± 37.57	158.5 ± 67.1	< 0.001^¶$€^
ESV (ml)	36.8 ± 18.6	36.4 ± 3.5	57.4 ± 36.4	98 ± 66.7	< 0.001^¶$€^
EF (%)	62.8 ± 8.7	67 ± 4.9	56.4 ± 14	44.1 ± 14.9	< 0.001^#¶$€^
e'-mean (cm/s)	9.8 ± 3.1	8.5 ± 4.1	7 ± 1.9	6 ± 1.9	< 0.001^#¶^
S’-mean (cm/s)	8.3 ± 1.8	8.6 ± 1.8	7.3 ± 1.9	5.8 ± 1.15	< 0.001^¶$^
E/A	1.1 ± 0.4	1.53 ± 0.73	0.98 ± 0.4	1.7 ± 0.87	< 0.001^¶€^
E/e'	7.9 ± 2.4	12.8 ± 5.4	12.2 ± 3.7	17.1 ± 5.3	< 0.001^#¶€^
PREp (mean), (cm/s)	6.9 ± 1.9	4.4 ± 0.97	5.9 ± 1.9	3.3 ± 1.1	< 0.001^*#¶€^
PREn (mean), (cm/s)	3.1 ± 0.9	4.6 ± 1.1	3.7 ± 1.3	3.8 ± 1.4	< 0.001^*#¶^
POEn (mean), (cm/s)	3.8 ± 1.1	3.2 ± 0.6	2.6 ± 0.9	2.2 ± 0.6	< 0.001^#¶^
POEp (mean), (cm/s)	2.3 ± 0.87	1.9 ± 0.5	2.97 ± 0.82	3.3 ± 0.9	< 0.001^#¶‡$€^
PREp /PREn (PRE-P/N ratio)	2.4 ± 0.97	0.98 ± 0.2	1.64 ± 0.56	0.9 ± 0.17	< 0.001^*#¶€^
POEn/POEp (POE-N/P ratio)	2.1 ± 1.1	1.7 ± 0.5	0.87 ± 0.19	0.68 ± 0.21	< 0.001^#¶^
PREp-mean/S’-mean	0.87 ± 0.3	0.5 ± 0.1	0.8 ± 0.2	0.61 ± 0.17	< 0.001^*¶^
QRS duration, ms (**)	79.5 ± 19.7	73.6 ± 3.1	87.4 ± 23.4	98.4 ± 23.2	0.028^*#^
LBBB, n(%)(**)	2(6%)	0(0%)	3(10%)	3(18%)	0.519
RBBB, n(%)(**)	1(3%)	0(0%)	2(7%)	0(0%)	0.626
IVCD, n(%)(**)	0(0%)	0(0%)	1(3%)	3(18%)	0.041

^*^ *p* < 0.05 between Normal and IC reversed

^#^ *P* < 0.05 Normal and IR reversed

^¶^ *p* < 0.05 between Normal and totally reversed

^‡^ *p* < 0.05 between IC reversed and IR reversed

^$^ *p* < 0.05 between IC reversed and totally reversed

^€^ *p* < 0.05 between IR reversed and totally reversed

(**) calculated from patients with heart failure

*PREp* positive pre-ejection, *PREn* negative pre-ejection, *POEn* negative post-ejection, *POEp* positive post-ejection *LBBB* left bundle branch block, *RBBB* right bundle branch block, *IVCD* intra-ventricular consuction delay

### Correlations for biphasic pre and post-ejection waves

**Fig. 3:**
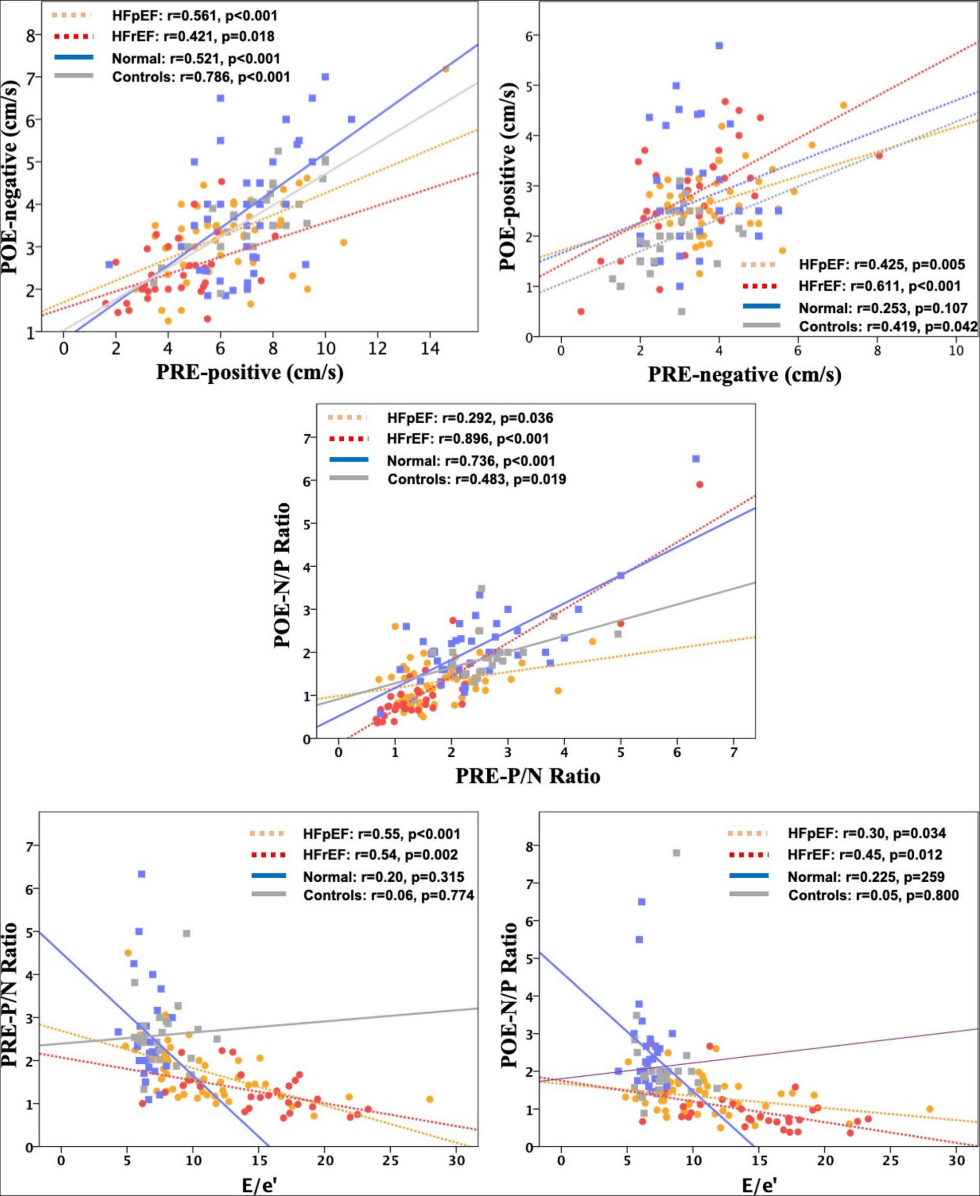
Dot plots for correlations observed in our study in subgroups. Upper panel: correlation between opposite waves of the biphasic PRE velocities (left: between positive PRE and negative POE, right: between negative PRE and positive POE). Middle panel: correlation between the ratio of positive to negative PRE waves (PRE-P/N) vs, the negative to positive POE (POE-N/P). Lower panel: correlations against E/e’ as a representation of LV filling pressures (left: versus the PRE-P/N ratio, right: versus the POE-N/P ratio)

As suggested by the concordance of the biphasic waves, the main waves in the normal pattern were the positively directed in PRE and negatively directed in POE. Correlations noted in our study between different PRE and POE velocities are summarized in Table 3 and Fig. 3. When correlation was checked between PREp and POEn it was found that both waves correlated significantly in all study subgroups, while the correlations were stronger in normal and matched controls (0.52, 0.79, respectively, all *p* < 0.001) and HFpEF (r = 0.56, *p* < 0.001), it was slightly weaker in patients with HFrEF (r = 0.42, *p* = 0.018). On the other hand, correlations between PREn and POEp were mainly noted in heart failure patients as no correlation was noted in normal subjects (r = 0.25, *p* = 0.107), and only a weak correlation was noted in matched controls (r = 0.42, *p* = 0.042). The correlation became slightly stronger in HFpEF (0.43, *p* = 0.005) and was strongest in HFrEF (r = 0.611, *p* < 0.001). As such it was noted that the pattern of correlation was opposite in controls and heart failure between signals associated with normal concordance and signals associated with reversed concordance showing the normal concordance signals correlate better in controls and reversed concordance signals correlate better in HF patients.

**Table 3:** Correlations observed in our study

	**HFpEF (*n* = 52)**		**HFrEF (*n* = 32)**		**Normal (*n* = 42)**		**Age matched Control (*n* = 26)**	
	**r**	**p**	**R**	**p**	**r**	**P**	**r**	**p**
**PREp (mean) vs. POEn (mean)**	0.561	< 0.001	0.421	0.018	0.521	< 0.001	0.786	< 0.001
**PREn (mean) vs. POEp (mean)**	0.425	0.005	0.611	< 0.001	0.253	0.107	0.419	0.042
**PREp/PREn (PRE-P/N) vs. POEn/POEp (POE-N/P)**	0.292	0.036	0.896	< 0.001	0.736	< 0.001	0.483	0.019
**Versus E/e’**								
**PREp (mean), (cm/s)**	0.513	< 0.001	0.4	0.025	0.04	0.843	0.02	0.928
**PREn (mean), (cm/s)**	0.07	0.609	0.144	0.439	0.177	0.377	0.06	0.770
**POEn (mean), (cm/s)**	0.432	0.002	0.256	0.165	0.142	0.481	0.04	0.853
**POEp (mean), (cm/s)**	0.136	0.342	0.363	0.045	0.174	0.385	0.042	0.844
**PREp/PREn (IC-P/N ratio)**	0.55	< 0.001	0.54	0.002	0.20	0.315	0.06	0.774
**POEn/POEp (IR-N/P ratio)**	0.3	0.034	0.45	0.012	0.225	0.259	0.05	0.800
**Versus EF**								
**PREp (mean), (cm/s)**	0.068	0.631	0.413	0.021	0.022	0.888	0.253	0.211
**PREn (mean), (cm/s)**	0.075	0.596	0.189	0.308	0.232	0.139	0.172	0.400
**POEn (mean), (cm/s)**	0.169	0.230	0.290	0.113	0.085	0.59	0.366	0.07
**POEp (mean), (cm/s)**	0.084	0.552	0.173	0.353	0.211	0.180	0.202	0.323
**PREp/PREn (IC-P/N ratio)**	0.116	0.414	0.230	0.222	0.225	0.152	0.103	0.631
**POEn/POEp (IR-N/P ratio)**	0.045	0.750	0.036	0.849	0.180	0.255	0.106	0.940

*PREp* positive pre-ejection*, PREn* negative pre-ejection*, POEn* negative post-ejection*, POEp* positive post-ejection

Correlation between the ratios PRE-P/N and POE-N/P, however, showed significant strong correlations in normal young subjects, weaker correlations in matched controls and HFpEF, while it was the strongest in HFrEF.

### Correlations of the pre and post-ejection waves versus E/e’ and EF

Correlations noted in our study against left ventricular filling pressures as expressed by E/e’ are summarized in Table 3 and Fig. 3. Importantly, it was shown that, most of the correlations of the biphasic velocities were noted in HF patients and not in controls. Briefly negative correlations were found between E/e’ and PREp in patients with HFpEF and HFrEF (r = -0.51, -0.4, *p* < 0.001, = 0.025), between E/e’ and POEn in patients with HFpEF only (r = -0.43, *p* = 0.002), between E/e’ and POEp in patients HFrEF only (r = -0.36, *p* = 0.045), while PREn did not correlate with E/e’ in any of the patients. The ratio PRE-P/N was found to correlate the strongest with E/e’ in both HFpEF and HFrEF (r = -0.55, -0.54; *p* < 0.001, = 0.002, respectively), while the ratio POE-N/P showed similar however weaker pattern of correlations (r = -0.3, -0.45, *p* = 0.034, 0.012, respectively).

**Fig. 4:**
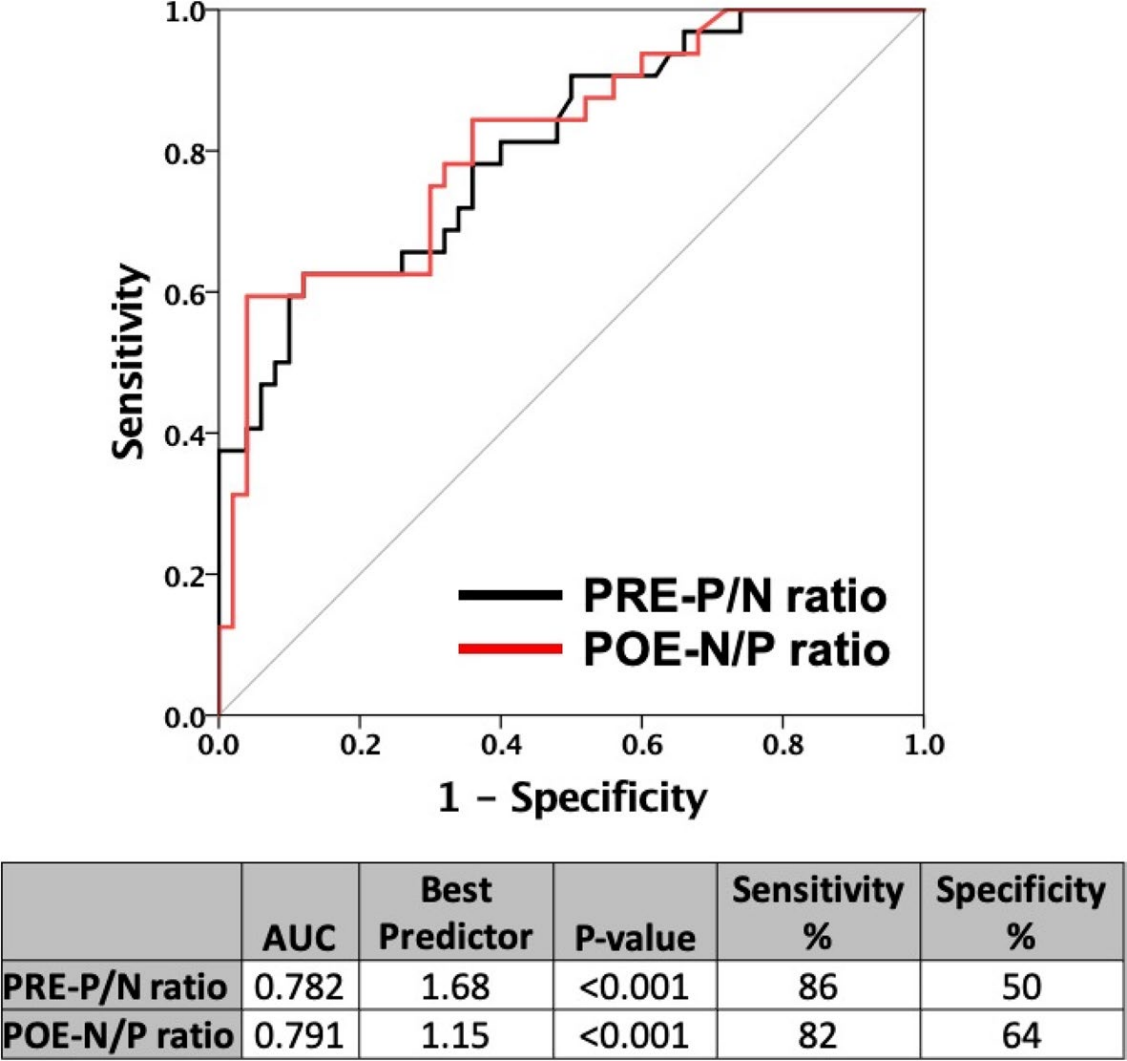
Receiver operator characteristic curves (ROC-curves) for best predictors of E/e’ ≥ 13 in patients with heart failure. Black line, positive PRE and negative PRE (PRE-P/N) ratio, red line, between negative POE to positive POE (POE-N/P) ratio

Receiver operator characteristic curves (Fig. 4) showed that the best predictors for E/e’ > 13 was PRE-P/N ≤ 1.68 (AUC:0.782, sensitivity: 86%, specificity 50%) while that for POE-N/P was ≤ 1.15 (AUC:0.791, sensitivity: 82%, specificity 64%).

Finally, correlations noted in our study against left ventricular EF are summarized in Table 3. The only variable that showed correlation with EF was PREp in patients with HFrEF (r = 0.413, *p* = 0.021). Other than that, PREp, PREn, POEn, and POEp velocities as well as the ratios PRE-P/N and POE-N/P did not correlate with EF in any of the groups of the study patients or control groups.

### Reproducibility

Reproducibility parameters are summarized in Table 4. For inter-observer variability, the absolute differences and interclass correlation coefficient for PREp were 0.03 ± 0.93 cm/s, 0.94, respectively, for PREn were 0.72 ± 0.67 cm/s, 0.84, respectively, for POEn were -0.53 ± 0.38 cm/s, 0.91, respectively, and for POEp were 0.01 ± 0.38 cm/s, 0.88, respectively. On the other hand, for intra-observer variability, the absolute differences and interclass correlation coefficient for PREp were 0.11 ± 0.57 cm/s, 0.97, respectively, for PREn were -0.22 ± 0.45 cm/s, 0.93, respectively, for POEn were -0.17 ± 0.31 cm/s, 0.93, respectively, and for POEp were 0.02 ± 0.34 cm/s, 0.89, respectively.

**Table 4:** Reproducibility analyses

	**Inter-observer variability**			**Intra-observer variability**		
	**ICC**	***p*-value**	**Difference**	**ICC**	***p*-value**	**Difference**
**PREp, cm/s**	0.94	< 0.001	0.03 ± 0.93	0.97	< 0.001	0.11 ± 0.57
**PREn, cm/s**	0.84	< 0.001	-0.72 ± 0.67	0.93	< 0.001	-0.22 ± 0.45
**POEn, cm/s**	0.91	< 0.001	-0.53 ± 0.38	0.93	< 0.001	-0.17 ± 0.31
**POEp, cm/s**	0.88	< 0.001	0.01 ± 0.38	0.89	< 0.001	0.02 ± 0.34

*PREp* positive pre-ejection, *PREn* negative pre-ejection, *POEn* negative post-ejection, *POEp* positive post-ejection

## Discussion

The main study findings are as follows: first, normally, tissue Doppler-derived biphasic signals are predominantly positive during pre-ejection (PRE) phase and predominantly negative during post-ejection (POE) phase. Second, normal concordance of both velocity signals (positive PRE and negative POE) is associated with the best clinical, hemodynamic, and functional profiles in heart failure patients. Partially reversed concordance (reversal of one of the velocity signals i.e. negative PRE and POE or positive PRE and POE) occurs in heart failure patients (both HFpEF and HFrEF) and is associated with an intermediate worsening of clinical and hemodynamic profiles. Completely reversed concordance (negative PRE and positive POE) occurs mostly in patients with HFrEF and is characterized by the worst clinical, hemodynamic, and functional profiles. Third, velocities of opposite directions during the pre-ejection and post-ejection phases exhibit positive correlations, and seem to be related to the LV filling pressures as expressed by E/e’ ratio.

### Myocardial mechanics during the pre-ejection and post ejection and their significance

The LV mechanical behavior during pre-ejection and post-ejection is a complex and controversial topic [8–10]. Pre-ejection is traditionally biphasic [11], the first phase is referred to as electromechanical delay (EMD) and is represented by the interval from the onset of the electrocardiographic QRS complex to mitral valve closure (MVC), and the second phase is referred to as isovolumic contraction (IC) which follows MVC and is characterized by a rapid increase in LV pressure before opening of the aortic valve. Recent studies have shown that two opposing mechanical behavior occur during the pre-ejection phase: subendocardial shortening exhibited during the first phase (EMD), accompanied by stretching of the subepicardial fibers during the second phase (IC) [12]. Studies have shown that this mechanical behavior can be mirrored by the biphasic mitral annular TDI-derived velocities[7], such that the positive component occurring during the EMD corresponds to the active subendocardial shortening and the negative component occurring during the IC corresponds to the passive subepicardial stretch. The significance of this complex mechanical behavior is reportedly tightly related to efficient systolic ejection [13]. The predominance of the positive component of the biphasic pre-ejection velocity in our study suggests that the prevailing and dominant force of active shortening over the passive stretch functions resulting in a higher corresponding positive over negative velocity. In our study, the reversal of this predominance (negative predominance of the PRE) may suggest loss of the magnitude of the active shortening, or a pathological prevalence of the stretch force associated with heart failure which may explain the worse systolic and diastolic functions.

Reportedly, the forces produced during pre-ejection are stored in memory within the myocardial wall until the end of the ejection phase [7, 14]. Reversal of the shortening-lengthening relationship defines the physiological onset of relaxation. In our study, opposite TDI velocities of both pre-ejection and post-ejection phases correlated positively, supporting the reversal of the shortening–stretch relationships from PRE into POE.

Importantly, the positive predominance of the PRE and the negative predominance of the POE suggests that the magnitude of the active forces (shortening) during PRE and the magnitude of their release during POE exceeds that exerted by the passive fiber stretch and its reversal and further suggest that the active endocardial mechanics are the driving force in all isovolumic mechanical behavior over the passive epicardial mechanics.

### Concordance and its effect on myocardial mechanics and functions

The coupling of normal predominance of both phases, noted in our study as the normal concordance between both phases, was mostly found in normal young patients and in age matched controls. Moreover, when such normal concordance was encountered in heart failure patients, it was associated with the best clinical and hemodynamic profile. The loss of normal concordance was almost always found in heart failure patients and was characterized by either reversal of one of both phases (partial reversal) or reversal of both phases (complete reversal). The progression from normal concordance to partially reversed and finally to completely reversed concordance in our study was associated with a similar progression in severity of the clinical and hemodynamic profiles in heart failure patients. Importantly, it was uncommon to have an isolated reversal of PRE (i.e. a reversed PRE without coupled reversal of the POE), while isolated reversal of the POE was not uncommon. Partial reversal of the predominance of the biphasic velocities may be due to failure of normal transmission of the pre-ejection forces (shortening–stretch) in memory into the post-ejection phase which may lead to inappropriate diastolic behaviors (as noted in patients with HFpEF) but may also affect systolic function to a certain degree. Complete reversal, on the other hand, means complete disruption of the presumed PRE-POE relationships and may be due to faulty generation of PRE forces and subsequent faulty transmission of these forces into the POE.

The mechanistic explanation of these changes lies in the fact that PRE-POE relationship is based on myocardial electromechanical anisotropic coupling [15, 16]. In normal physiological situations, the electrical activation occurs in an endocardial to epicardial direction and begins subendocardially near the apical septum and spreads rapidly toward the base. The rapid apico-basal spread of electrical activation within the subendocardium initiates the contraction sequence and coincides with an early rapid build-up of intraventricular pressure during the pre-ejection period. Subepicardial deformation occurs later temporally coinciding with the onset of systolic ejection. Importantly, this apico-basal wave of endocardial shortening aids in milking blood towards the LV outflow. Conversely, during repolarization, transmural electrical gradients propagate in a base-to-apex direction. As such, mechanical coupling during post-ejection (reversal of the shortening-lengthening forces) follows the same baso-apical direction aiding in formation of suction pressure and early diastolic filling.

This timing and sequence of electrical excitation influenced by the His-Purkinje system and the anistropic nature of myocardium [16], both of which are prone to disruption in heart failure patients as a result of microscopic fibrosis or loss of gap junctions. In our study, this hypothesis may be suggested by the fact the occurrence of intraventricular conduction delay was associated with reversal of velocities especially with totally reversed concordance, and similarly, QRS duration was prolonged the most in those patients. As such this electrical disruption may lead to faulty initiation, and/or transition of mechanical forces between pre-ejection and post-ejection and loss of mechanical coupling expressed as the shortening-lengthening forces and thus may explain the functional and hemodynamic outcomes of reversal of predominance and concordance of pre- and post-ejection velocities in our study.

### Limitations

The study suffered from the following limitations: first, the sample size is small and larger studies should be conducted before the findings can be adopted to clinical practice. Second, mitral annular velocities are the result of the tethering of the fibrous structure of the mitral annulus, and as such represents only an indirect measure of myocardial mechanics. Better representation of the myocardial mechanics can be obtained using myocardial deformation imaging techniques such as speckle tracking echocardiography. Strain rate imaging would be an appropriate alternative, however, due to the lower frame rate and higher signal to noise ratio, deformations during PRE and POE can either be missed or recorded with high noise, making it less reproducible than spectral tissue Doppler of the mitral annulus. Third, mitral annular velocities represent mainly deformations in the longitudinal directions, however deformations during pre- and post-ejection phases occur in all other directions of contraction, namely, circumferential, radial, as well as rotational (twist). Further studies should focus and compare the relationships of deformation in other directions of myocardial contraction in PRE and POE both in health and disease. Forth, reduced EF in our study was defined as EF ≤ 50% according to the American society of echocardiography guidelines for chamber quantification [17]. It is important to note that recently a separate category of patients with EF between 40–50% is thought to be categorized as patients with mid-range EF (HFmrEF). In our study we have not studied HFmrEF as a separate category as we were more focused on changes with any EF abnormality. It is important to note that this catogery of patients represent a controversy as to which category of HF it behaves similarly. Recently, evidence suggested regression towards including these patients as HFrEF with mild reduction of systolic function as it was found to be behaving similarly [18]. As such, in our study, we have included this subcategory of patients as patients with HFrEF. Fifth, the status of heart failure in these patients was clinically assessed using NYHA functional class. Brain natriuretic peptide (BNP) is reportedly a more objective way to assess heart failure, however it was not measured in our study. In our patients, echocardiograms were done on an outpatient basis, and as such BNP was not measured in relation to the echocardiographic studies. However, the diagnosis of these patients was previously established prior to their echocardiographic studies according to the guidelines using clinical presentation and laboratory findings including BNP. Finally, the relationships to systolic and diastolic functions lack invasive reference methods for comparison, further studies should consider reproduction of the correlations observed against invasively measured pressures.

## Conclusions

Tissue Doppler-derived biphasic velocities during the pre- and post-ejection phases can be related to the active endocardial shortening and passive epicardial stretch forces that occur during PRE and their reversal during POE. In normal physiological state, biphasic signals are predominantly positive during PRE and predominantly negative during POE highlighting the prevalence of the active endocardial shortening over the passive epicardial stretch forces. A positive correlation between the opposite velocities of PRE and POE represent the PRE-generation and POE-reversal of shortening–stretch relationships, the attenuation of which may be related to elevated LV filling pressures in patients with heart failure as expressed by E/e’ ratio. In heart failure patients, a normal concordance of both velocity signals is associated with the best clinical, hemodynamic profiles, while partially reversed (either PRE or POE), and completely reversed concordance are associated with progressive worsening of clinical and hemodynamic profiles. Reversal of concordance can be explained by faulty generation and/or propagation of shortening–stretch forces due to mechanical barriers such as fibrosis or electrical abnormalities such as loss of gap junctions that occur in the myocardium of patients with heart failure. The relationships noted in our study can aid in the prediction of the prognosis in heart failure patients and may aid therapeutic selection and dosing, however larger studies are needed to test the clinical implications of such findings.

## Data Availability

The datasets used and/or analysed during the current study are available from the corresponding author on reasonable request.

## References

[1] Mosterd A, Hoes AW (2007). Clinical epidemiology of heart failure. Heart.

[2] Omar AM, Bansal M, Sengupta PP (2016). Advances in Echocardiographic Imaging in Heart Failure With Reduced and Preserved Ejection Fraction. Circ Res.

[3] Cho EJ, Caracciolo G, Khandheria BK, Steidley DE, Scott R, Abhayaratna WP, Chandrasekaran K, Sengupta PP (2010). Tissue Doppler image-derived measurements during isovolumic contraction predict exercise capacity in patients with reduced left ventricular ejection fraction. JACC Cardiovasc Imaging.

[4] Lindqvist P, Waldenstrom A, Wikstrom G, Kazzam E (2007). Potential use of isovolumic contraction velocity in assessment of left ventricular contractility in man: A simultaneous pulsed Doppler tissue imaging and cardiac catheterization study. Eur J Echocardiogr.

[5] Salem Omar AM, Tanaka H, Matsumoto K, Tatsumi K, Miyoshi T, Hiraishi M, Tsuji T, Kaneko A, Ryo K, Fukuda Y (2012). Tissue Doppler imaging-derived myocardial acceleration during isovolumetric contraction predicts pulmonary capillary wedge pressure in patients with reduced ejection fraction. Circ J.

[6] Omar AM, Abdel-Rahman MA, Khorshid H, Helmy M, Raslan H, Rifaie O (2015). Tissue Doppler-Derived Myocardial Acceleration during Isovolumetric Contraction Predicts Pulmonary Capillary Wedge Pressure in Patients with Significant Mitral Regurgitation. Ultrasound Med Biol.

[7] Sengupta PP, Khandheria BK, Korinek J, Wang J, Belohlavek M (2005). Biphasic tissue Doppler waveforms during isovolumic phases are associated with asynchronous deformation of subendocardial and subepicardial layers. J Appl Physiol  (1985).

[8] Omar AMS, Ronderos Botero DM, Arreaza Caraballo J, Kim GH, Khachatoorian Y, Sharma P, Bella JN, Contreras J, Rifaie O, Abdel-Rahman MA (2021). Combined atrioventricular longitudinal strain rate during isovolumic contraction predicts pulmonary capillary wedge pressure in patients with systolic dysfunction. Am J Cardiovasc Dis.

[9] Stoylen A, Daae AS (2021). Physiological significance of pre- and post-ejection left ventricular tissue velocities and relations to mitral and aortic valve closures. Clin Physiol Funct Imaging.

[10] Vancheri F, Henein M (2018). The impact of age on cardiac electromechanical function in asymptomatic individuals. Echocardiography.

[11] Remme EW, Lyseggen E, Helle-Valle T, Opdahl A, Pettersen E, Vartdal T, Ragnarsson A, Ljosland M, Ihlen H, Edvardsen T (2008). Mechanisms of preejection and postejection velocity spikes in left ventricular myocardium: interaction between wall deformation and valve events. Circulation.

[12] Sengupta PP: Exploring left ventricular isovolumic shortening and stretch mechanics: "The heart has its reasons...". JACC Cardiovasc Imaging 2009, 2(2):212–215. 10.1016/j.jcmg.2008.12.005 19356558

[13] Campbell KB, Chandra M (2006). Functions of stretch activation in heart muscle. J Gen Physiol.

[14] Sengupta PP, Khandheria BK, Korinek J, Wang J, Jahangir A, Seward JB, Belohlavek M (2006). Apex-to-base dispersion in regional timing of left ventricular shortening and lengthening. J Am Coll Cardiol.

[15] Durrer D, van Dam RT, Freud GE, Janse MJ, Meijler FL, Arzbaecher RC (1970). Total excitation of the isolated human heart. Circulation.

[16] Sengupta PP, Tondato F, Khandheria BK, Belohlavek M, Jahangir A (2008). Electromechanical activation sequence in normal heart. Heart Fail Clin.

[17] Lang RM, Badano LP, Mor-Avi V, Afilalo J, Armstrong A, Ernande L, Flachskampf FA, Foster E, Goldstein SA, Kuznetsova T (2015). Recommendations for cardiac chamber quantification by echocardiography in adults: an update from the American Society of Echocardiography and the European Association of Cardiovascular Imaging. J Am Soc Echocardiogr.

[18] Savarese G, Stolfo D, Sinagra G, Lund LH (2022). Heart failure with mid-range or mildly reduced ejection fraction. Nat Rev Cardiol.

